# HBP-net for robust remote heart rate estimation using heartbeat probability

**DOI:** 10.1016/j.isci.2026.114974

**Published:** 2026-02-11

**Authors:** Xiaolang Ye, Caiying Zhou, Yuanwang Wei, Fried-Michael Dahlweid, Hong Sun, Chaochao Wang, Xianchao Zhang

**Affiliations:** 1 Provincial Key Laboratory of Multimodal Perceiving and Intelligent Systems, Jiaxing University, Jiaxing, Zhejiang 314001, China; 2 College of Science, Jiangxi University of Science and Technology, Ganzhou, Jiangxi 341000, China; 3 Key Laboratory of Medical Electronics and Digital Health of Zhejiang Province, Jiaxing University, Jiaxing, Zhejiang 314001, China; 4 Engineering Research Center of Intelligent Human Health Situation Awareness of Zhejiang Province, Jiaxing University, Jiaxing, Zhejiang 314001, China; 5 Institute of Information Network & Artificial Intelligence, Jiaxing University, Jiaxing, Zhejiang 314001, China; 6 Dedalus Healthcare, Milan 20121, Italy; 7 Sino-European Joint Lab for Health Information Processing and Applications, Jiaxing University, Jiaxing, Zhejiang 314001, China

**Keywords:** Health sciences

## Abstract

Remote photoplethysmography (rPPG) enables contactless heart rate monitoring but remains vulnerable to motion and lighting changes. We address this by reframing heart rate estimation as a heartbeat detection problem, bypassing the need to reconstruct full blood volume pulse signals. Our approach, HBP-Net, predicts heartbeat probability directly from facial video using a spatiotemporal attention architecture, improving robustness while reducing computational complexity. Evaluated across multiple datasets—including a new motion-challenged benchmark—HBP-Net achieves competitive accuracy under static conditions and maintains performance as motion increases. This shift from signal reconstruction to probabilistic event detection offers a conceptually simpler and more resilient framework for rPPG. The method advances the feasibility of reliable, camera-based vital sign monitoring in real-world settings such as telehealth, fitness tracking, and continuous patient assessment.

## Highlights

• Introduces a probabilistic approach for robust remote heart rate estimation
• Achieves reliable performance under real-world motion and lighting changes
• Demonstrates state-of-the-art performance on motion-intensive benchmark datasets
• Releases open-source code and a unified evaluation framework for the community

## Graphical abstract

**Figure:**
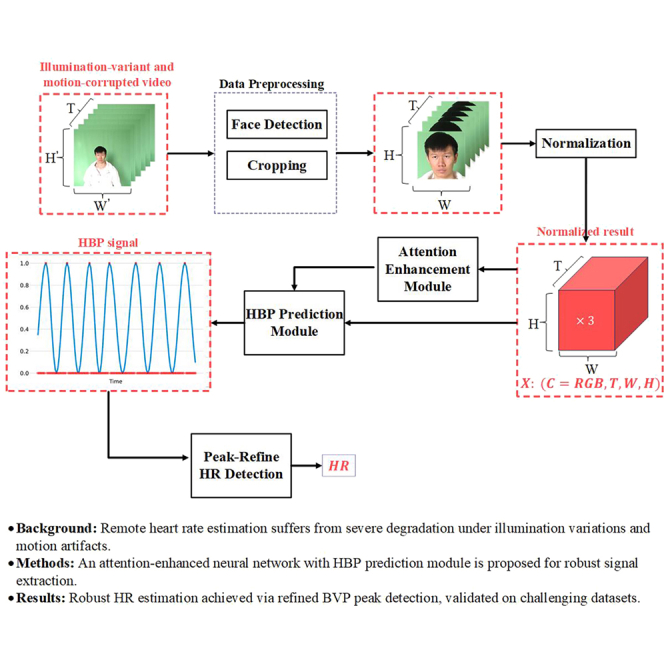

## Introduction

Heart rate (HR) is a key physiological indicator for assessing cardiovascular health, emotional states, and physical load. Due to its wide applications in healthcare, sports management, and emotion detection, heart rate monitoring technology continues to attract attention from both academia and industry. Traditional heart rate monitoring methods primarily include photoplethysmography (PPG) and electrocardiography (ECG). PPG technology detects pulsatile blood flow by measuring changes in light reflectance or transmittance at the skin surface and is commonly used in wearable devices.^1^ On the other hand, ECG monitors heart rate by detecting voltage changes generated by myocardial electrical activity and is widely used in clinical settings.^2^ However, these traditional methods have limitations, as they primarily rely on contact sensors that require direct skin contact. This can cause discomfort during prolonged wear and render the signal susceptible to motion artifacts.^3^ Additionally, device fragility and user compliance further limit their widespread application. To overcome these issues, both academia and industry have begun exploring non-contact heart rate monitoring technologies.^4^^,^^5^

In recent years, remote photoplethysmography (rPPG) has emerged as a non-invasive, convenient, and adaptable heart rate monitoring technology that has garnered significant attention. The feasibility of rPPG was first demonstrated in 2008 by Verkruysse et al.,^6^ who showed that subtle skin color variations induced by blood volume pulsations (BVPs) could be captured by an ordinary camera, thereby establishing the foundation for remote physiological sensing. By analyzing these hemodynamic changes from facial videos, rPPG enables the remote estimation of heart rate without physical contact. Compared to contact-based methods, rPPG offers advantages such as non-invasiveness, convenience, and adaptability to various scenarios, making it particularly suitable for smart health monitoring, remote health management, and physiological monitoring in challenging environments.^7^^,^^8^^,^^9^

Early rPPG approaches relied on handcrafted signal processing algorithms. Poh et al.^10^^,^^11^ introduced independent component analysis (ICA) to decompose RGB signals, significantly improving robustness against ambient lighting changes. Subsequent methods further refined signal extraction: the CHROM algorithm^12^ optimized color channel ratios to suppress illumination artifacts, while the POS method^13^ employed linear combinations in chrominance space to enhance stability under motion and varying lighting. Despite their success in controlled settings, these traditional approaches rely on fixed signal models and demonstrate poor noise robustness—especially under variations in lighting or interference from motion artifacts.^14^^,^^15^

The rise of deep learning has catalyzed a paradigm shift in rPPG, enabling end-to-end learning of physiological features directly from video. Early deep models leveraged 2D CNNs for spatial region of interest (ROI) modeling,^16^ but soon evolved toward spatiotemporal architectures. PhysNet^17^ pioneered the use of 3D convolutions within an encoder-decoder framework to capture dynamic BVP patterns across time and space. Follow-up works enhanced this architecture with multi-scale features^18^ or adversarial training^19^ to better separate physiological signals from noise. Concurrently, recurrent structures such as LSTMs were integrated to model temporal dependencies,^20^^,^^21^ further boosting performance under motion. More recently, attention mechanisms have been incorporated to guide models toward physiologically relevant regions. Methods such as DeepPhys,^22^ MTTS-CAN,^23^ and LSTC-rPPG^16^ use spatial or channel attention to dynamically weight informative pixels or features. This trend culminated in designs such as the information-enhanced network,^24^ which fuses local and global context to improve robustness, and the Multi-scale Spatial-Temporal Decomposition Network (MSDN),^25^ which disentangles motion and physiological components across scales. In parallel, spatiotemporal Convolutional Networks have gained increasing popularity, leveraging 3D convolution to effectively capture spatiotemporal information and enhance the accuracy of heart rate estimation.^26^ These deep learning approaches—such as convolutional neural networks (CNNs) and long short-term memory networks (LSTMs)—have significantly improved heart rate estimation under varying lighting conditions and complex backgrounds.^27^

Despite these advances, current rPPG methods predominantly frame heart rate estimation as a regression or BVP signal reconstruction task, requiring post-processing (e.g., peak detection) to derive final heart rates. Moreover, most studies focus on extracting BVP signals from facial videos to estimate heart rate. However, from the perspective of heart rate calculation, directly extracting BVP signals is not always necessary. Our experimental results demonstrate that directly predicting the probability of a heartbeat represents a more effective approach for obtaining heart rate or heart rate variability, with enhanced robustness against noise interference. Particularly in motion scenarios, existing rPPG methods often face challenges due to artifacts caused by head and facial movements, which can degrade the accuracy of heart rate estimation. Although some efforts have been made—such as stabilizing video frames or removing background noise to mitigate the impact of motion artifacts,^8^ or improving model generalization through data augmentation or transfer learning^28^^,^^29^—these approaches still struggle to achieve accurate heart rate estimation in environments with significant motion interference. Consequently, establishing a stable method for extracting heart rate signals in motion scenarios remains a key challenge in the field of rPPG.^30^ Notably, due to data access limitations, many recent methods are evaluated only on specific benchmarks (e.g., PURE), and their generalizability across datasets—such as COHFACE or UBFC-rPPG—remains limited.

To address these challenges, we propose HBP-Net, a novel framework that reformulates rPPG as a heartbeat probability prediction task rather than a signal reconstruction problem. The main contributions of this article are as follows.

1. HBP-Net innovatively transforms the traditional sequence-to-sequence deep learning framework into a classification task, effectively reducing interference from ambient light variations and motion artifacts, thereby enabling robust and efficient heart rate estimation in complex scenarios.
2. HBP-Net introduces an innovative signal enhancement mechanism that significantly improves robustness against complex motion artifacts. This mechanism enhances facial BVP signals and facilitates their visualization, providing a more reliable feature representation for remote heart rate estimation.
3. HBP-Net demonstrates outstanding performance across multiple datasets, including ZJXU-MOTION,^31^ achieving superior heart rate estimation accuracy in motion scenarios. These results validate the robustness and adaptability of the proposed framework in challenging environments.

In summary, existing deep rPPG methods predominantly frame heart rate estimation as a regression or signal reconstruction task, requiring post-processing (e.g., peak detection) to derive final heart rates. In contrast, our HBP-Net reformulates the problem as a heartbeat probability prediction task, enabling direct, end-to-end heart rate computation without intermediate signal reconstruction or heuristic post-processing. This design not only simplifies the pipeline but also reduces computational overhead while maintaining high accuracy—offering a more efficient and robust alternative for real-world deployment.

### Proposed method

The proposed method operates as follows: First, the input video clips are segmented into fixed-length time windows, and the corresponding BVP signals are transformed into a Heartbeat Probability (HBP) distribution to serve as the model’s label. This preprocessed data is then fed into HBP-Net, a core architecture built upon a skip-connected 3D encoder-decoder designed to enhance BVP-related features across space and time. The network output is further processed by spatial convolutions to generate the final HBP map. The entire model is trained end-to-end using a binary cross-entropy loss function. Finally, the predicted HBP is used by an optimized peak detection algorithm to accurately determine the heart rate, significantly improving detection performance.

### Data preprocessing

**Figure 1:**
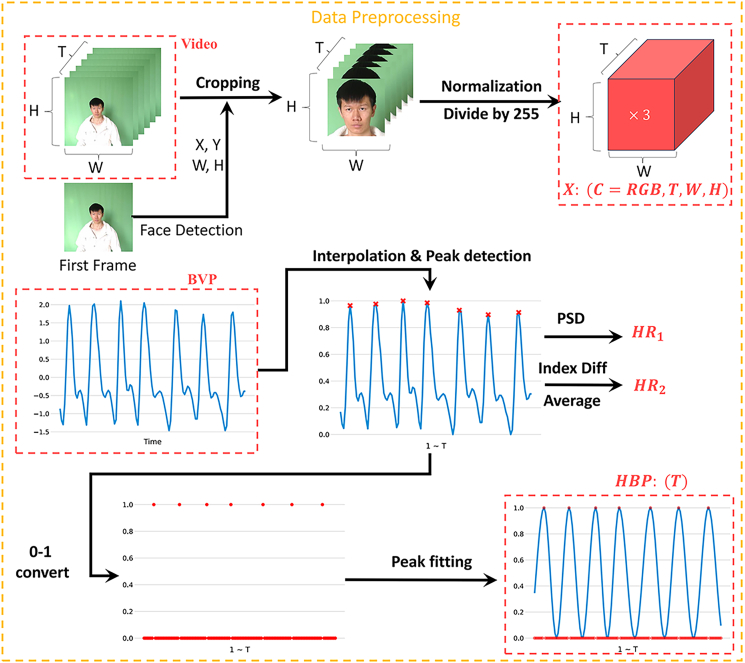
This flowchart outlines the end-to-end pipeline for video-based heart rate estimation, starting from raw video input and culminating in the generation of a probability distribution over possible heart rates Key stages include video segmentation, feature extraction, heart rate estimation, and the derivation of the final probability distribution.

After data preprocessing, the region of interest (ROI) is selected, and PPG or ECG signals are converted into HBP distributions, as illustrated in Figure 1. The input to the model consists of video sequences with a temporal length of T, where each frame is represented in RGB format. The video sequence is denoted as $X\in R^{B\times C\times T\times H\times W}$, where C represents the color channel, W is the frame width, and H is the frame height. We process the input video with dimensions (3,160,128,128). To balance computational efficiency and enable the model to capture contextual information through training, we employ MTCNN^32^ for face detection, extract facial regions from each frame, and normalize them to facilitate the model’s learning of subtle changes caused by cardiac pulses.

**Figure 2:**
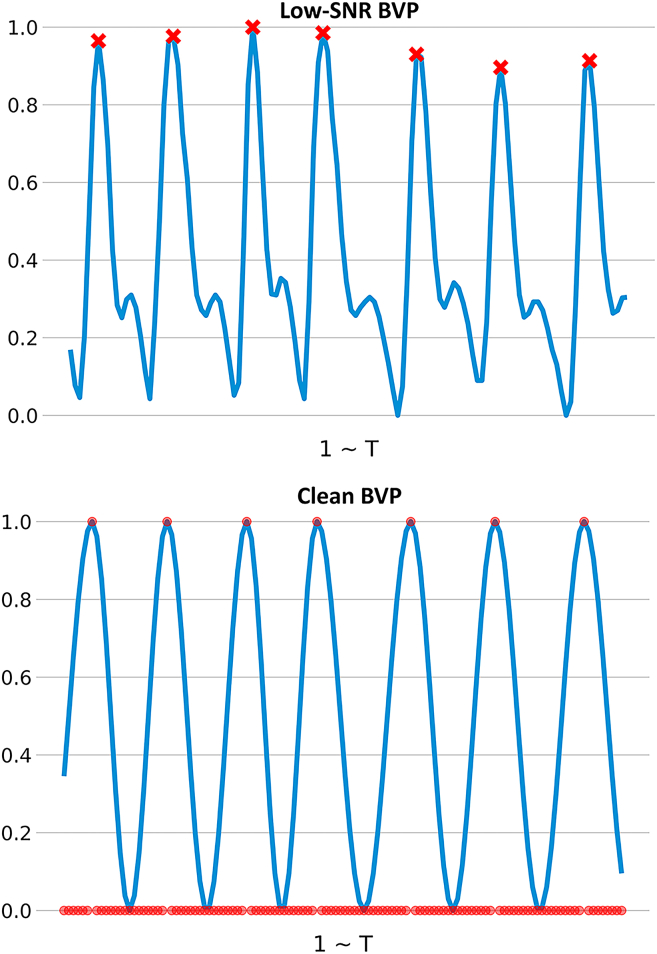
Comparison of clean and low-SNR BVP signals and their corresponding HBP maps, where T denotes the total number of time steps

The model’s labels are a sequence of HBP distributions ranging from 0 to 1. The BVP sequence undergoes linear interpolation and mean normalization to obtain a sequence of length T. Next, peak detection is performed, considering that BVP measurements may be affected by motion artifacts. Peaks are discarded if the heart rate derived from peak detection significantly differs from that obtained using the power spectral density (PSD) method. Let $P_{n}$ denote the peak index sequence of the BVP signal, and let $D_{\text{max}}=\max\left(\text{diff}\left(P\right)\right)$ represent the maximum interval between consecutive peaks, where diff denotes the first-order difference of the sequence. PB(x) and PF(x) denote the indices of the previous and following peaks relative to position x, respectively. The HBP is calculated using Equation 1, which aims to ensure that peak responses exhibit smooth decay rather than appearing as isolated impulses. For example, in image keypoint detection, a Gaussian function is often applied to make the probability distribution around the keypoint decrease smoothly. To illustrate the robustness of the probabilistic formulation under noise, we compare clean and low-SNR BVP signals along with their corresponding HBP maps in Figure 2.

(Equation 1) $${\text{HBP}}_{x}=\frac{1}{2}+\frac{1}{2}\times \left\{\begin{array}{cc} \cos\left(\frac{2\pi }{D_{\text{max}}}\cdot \left(x+D_{\text{max}}-P_{0}\right)\right), & x\in \left(0,P_{0}\right)\\ \cos\left(\frac{2\pi }{PF\left(x\right)-PB\left(x\right)}\cdot \left(x-PB\left(x\right)\right)\right), & x\in \left[P_{0},P_{T}\right]\\ \cos\left(\frac{2\pi }{D_{\text{max}}}\cdot \left(x-PB\left(x\right)\right)\right), & x\in \left(P_{T},T\right) \end{array}\right)$$

Replacing the full-BVP target with a smooth cosine-decay HBP map gives the network local, low-curvature supervision that is easier to fit and remains reliable when RGB frames are blurred or poorly lit; the paired attention module further amplifies subtle pulse features while suppressing motion artifacts, yielding joint input-output robustness without extra thresholds.

#### HBP-net model

**Figure 3:**
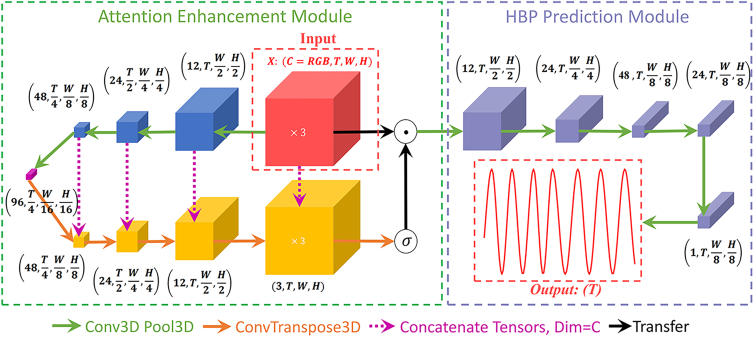
HBP-Net architecture: The overall structure of the HBP-Net is illustrated, where X denotes the input The left side of the diagram highlights the attention enhancement mechanism, while the right side depicts the downsampling process that generates the final Heart Beat Profile (HBP) output.

The framework of HBP-Net is illustrated in Figure 3. The architecture consists of two core modules: the attention enhancement module (AEM) and the HBP prediction module. The input video sequence X is first processed by the AEM to amplify the blood volume pulse (BVP) signal through spatiotemporal attention mechanisms. This enhanced signal is then fed into the HBP prediction module, which employs a hierarchical encoder-decoder structure with 3D convolutions, pooling, and skip connections to progressively compress spatial dimensions while preserving temporal information. The encoder reduces the spatial resolution from (H,W) to $\left(\frac{H}{8},\frac{W}{8}\right)$ across multiple stages, while the decoder gradually restores spatial detail through upsampling operations. Finally, the HBP predictor estimates the target distribution from the decoded features. The detailed architecture of the proposed HBP-Net is summarized in Table 1.

**Table 1:** The architecture of HBP-Net consists of a stack of N Conv3D layers, each followed by batch normalization (BN)

Input	Layers	Dimension	Layers	Output
X	Conv3d×12, BN, ReLU	[12, 128, 128, 128]	MaxPool3d(1, 2, 2)	Enc1: [12, 128, 64, 64]
Enc1	Conv3d×24, BN, ReLU	[24, 128, 64, 64]	MaxPool3d(2, 2, 2)	Enc2: [24, 64, 32, 32]
Enc2	Conv3d×48, BN, ReLU	[48, 64, 32, 32]	MaxPool3d(2, 2, 2)	Enc3: [48, 32, 16, 16]
Enc3	Conv3d×96, BN, ReLU	[96, 32, 16, 16]	MaxPool3d(1, 2, 2)	Enc4: [96, 32, 8, 8]
Enc4	ConvTranspose3d×48 + Enc3	[96, 32, 16, 16]	Conv3d×48, BN, ReLU	Dec4: [48, 32, 16, 16]
Dec4	ConvTranspose3d×24 + Enc2	[48, 64, 32, 32]	Conv3d×24, BN, ReLU	Dec3: [24, 64, 32, 32]
Dec3	ConvTranspose3d×12 + Enc1	[24, 128, 64, 64]	Conv3d×12, BN, ReLU	Dec2: [12, 128, 64, 64]
Dec2	ConvTranspose3d×3 + X	[12, 128, 128, 128]	Conv3d×3, BN, Sigmoid	Dec1: [3, 128, 128, 128]
Dec1	⊙X, Conv3d×12, BN, ReLU	[12, 128, 64, 64]	Conv3d×24, BN, ReLU	Out2: [24, 128, 32, 32]
Out2	Conv3d×48, BN, ReLU	[48, 128, 16, 16]	Conv3d×24, Conv3d×1	Out1: [1, 128, 16, 16]
Out1	AvgPool3d(128, 1, 1)	[1, 128, 1, 1]	Reshape(128)	Out: [128]

Here, each Conv3D layer applies a set of learnable 3D filters (the number of which is specified per layer), and element-wise multiplication is denoted by ⊙.

In the HBP-Net model, each frame of the RGB video is treated as a sampling point, and the model is trained to determine whether a particular frame represents a heartbeat. These frames collectively form a random process HBP(t), where the probability at any given time is correlated with both preceding and succeeding points. Consequently, knowing the signal values around a specific time point increases the likelihood of correctly predicting the signal at that moment. For example, detecting only the rise in BVP signal intensity does not determine the phase of the cardiac cycle. However, when the BVP signal increases and then decreases, there is a high probability that a heartbeat has occurred. Thus, the primary task of our model is to fit this probability distribution HBP(t). During training, the model learns the relationship between these changes and the occurrence of heartbeats, outputting the probability distribution of whether the frames in the image sequence correspond to heartbeats.

Spatiotemporal convolutions effectively capture these temporal and spatial correlations. However, experiments reveal that when the video contains significant noise, the attention mechanism of standard neural networks exhibits poor convergence. To address this challenge, we design the attention enhancement module (AEM) using a fully convolutional architecture with skip connections to enhance the BVP signal. The model is first trained on datasets with lower noise levels and is subsequently adapted to increasingly noisy datasets. The AEM computes attention weights through the following steps:

(Equation 2) $$A_{st}=\sigma \left(W_{st}\cdot \text{Conv}3\text{D}\left(X\right)\right)\in R^{B\times C\times T\times W\times H}$$

Here, $W_{st}$ represents the learned weights, Conv3D(X) extracts spatiotemporal features, and $A_{st}\in R^{B\times C\times T\times H\times W}$ is the spatiotemporal attention weight.

Next, the weighted feature map is computed using Equation 3.

(Equation 3) $$X^{*}=X\cdot A_{st}$$

Finally, $X^{*}$ is spatially compressed and pooled to output the HBP curve using Equation 4.

(Equation 4) $$\text{HBP}=\text{AvgPool}3\text{D}\left(\text{Conv}3\text{D}\left(X^{*}\right)\right)$$

This design prioritizes computational efficiency and task-specific feature extraction, leveraging localized operations for physiological signal estimation rather than global attention mechanisms, which would introduce unnecessary complexity for this application. The skip connections ensure multi-scale feature fusion, maintaining fine-grained details critical for accurate BVP estimation. While advanced architectures such as transformers, offer potential benefits, the current structure achieves an optimal balance between performance and efficiency for the target task. Future work could explore hybrid approaches to further enhance long-range dependency modeling without compromising real-time processing capabilities.

Although the most straightforward approach to heart rate estimation is to have the network directly regress a scalar HR value, we instead explicitly model the HBP distribution for three practical reasons.

##### Reducing learning difficulty

Directly regressing a single HR value from facial videos—especially under varying illumination and motion artifacts—is a challenging task that often leads to unstable training and poor generalization. By introducing the HBP map as an intermediate representation, the model can learn more fine-grained physiological features related to cardiac activity. This not only simplifies the learning task but also improves robustness against noise and enhances convergence stability.

##### Preserving extensibility

Unlike a scalar HR output, the HBP map retains coarse-grained temporal heartbeat information. While the current work focuses on heart rate estimation and does not yet achieve the millisecond-level precision required for reliable HRV analysis, this representation lays the groundwork for future extensions. It makes it possible to explore the estimation of RR intervals and other cardiovascular metrics without structural changes to the network, whereas a direct HR regression model would need to be retrained or redesigned for such tasks.

##### Robustness to noise

By learning a probabilistic distribution, the model can better handle noisy or missing peaks, which are common in motion scenarios. Direct HR regression may struggle with such variability.

#### Loss function

Unlike prior work that employs complex fused losses, we use standard binary cross-entropy (BCE) to directly supervise the peak-probability output. Equation 5 gives the standard BCE form used for training.

(Equation 5) $$L_{\text{train}}=\frac{1}{T}\overset{T}{\underset{i=1}{\sum }}\left[y_{i}\log{\hat{y}}_{i}+\left(1-y_{i}\right)\log\left(1-{\hat{y}}_{i}\right)\right]$$

Where:

• y: True probability distribution,
• $\hat{y}$: Predicted probability distribution,
• T: Number of samples.

We use standard binary cross-entropy; the previous subtraction of BCE(y,y) was removed as it is constant w.r.t. parameters and does not affect gradients.

**Figure 4:**
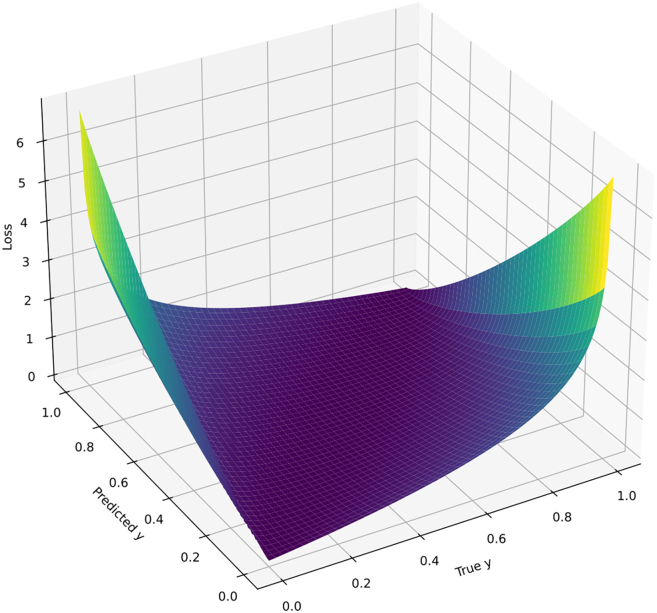
3D loss surface over true and predicted values: A three-dimensional surface plot depicts how the loss magnitude varies with respect to the ground-truth and predicted values

The second term of the subtraction BCE(y,y) does not contribute to gradient computation or backpropagation. The loss equals 0 if and only if $y=\hat{y}$. A 3D visualization of the loss function as a function of true values (y) and predicted values $\hat{y}$ is shown in Figure 4. When $y=\hat{y}$, the loss is 0. When $|y-\hat{y}|\to 1$, the loss approaches infinity. However, during computation, the loss is bounded at a maximum value of 10.

#### Peak-refine HR detection

**Figure 5:**
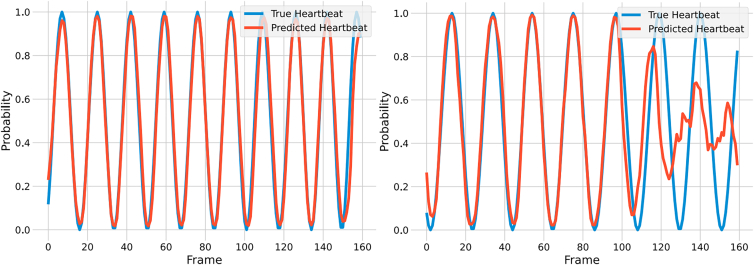
Successful vs. disrupted heartbeat predictions: The left plot illustrates successful heartbeat predictions with high-confidence detections, while the right plot shows disrupted predictions containing low-probability, noisy heartbeat sections

In existing literature, heart-rate detection from BVP often relies on peak detection or PSD. HBP-Net outputs a probability distribution (Figure 5). High-SNR segments (left plot) yield reliable peaks for both methods; low-SNR segments (right plot) render them unreliable, whereas the HBP curve still exhibits discernible local maxima.

Using the probability distribution from the HBP-Net model, we can develop a peak filtering method to measure a reliable heart rate. This method’s pseudocode is Algorithm 1.

Algorithm 1 **Peak-Refine HR**

**Input:** X: HBP array, confidence: float constant

**Output:** HR

**Function** detect*(X, confidence)***:**

 peaks← find_peaks(*X*);

 peaks_space_list← [ ];

 peak_index_before← None;

 **foreach** peak_index∈peaks **do**

 **if** peak_index_before **is** None **or**X[peak_index]>confidence **then**

 peak_index_before←peak_index;

 **continue**;

 
**end**

 **if** X[peak_index]≤confidence **then**

 peak_index_before←None;

 **continue**;

 
**end**

 peaks_space_list.append(peak_index−peak_index_before);

 
**end**

 peaks_space_list←median_filter(peaks_space_list);

 HR←fps/ mean(peaks_space_list) ×60;

 **return** *HR*;

Assuming that when the actual peaks are less affected by noise and only minor peaks exist, the confidence threshold will filter out these peaks, thereby improving accuracy. However, when the actual peaks are heavily noise-corrupted and no clear peaks are present, applying the confidence threshold may lead to the exclusion of these true peaks. Skipping such peaks and calculating the inter-peak intervals could significantly increase the error in heart rate estimation. Excessively large peak-to-peak intervals can be filtered out using moving median filtering to further reduce estimation errors.

Peak detection confidence is not fixed: for each dataset, we perform a grid search over the interval [0.6, 0.9] with a step size of 0.1 on the validation split and adopt the value yielding the lowest MAE. The exact confidence threshold is automatically determined without manual tuning.

## Results

In the Result Section, we conducted two sets of performance comparison experiments. In Sec.h, conventional evaluation methods were employed. All experiments were conducted within a single dataset, and three commonly used metrics were calculated for assessment: mean absolute error (MAE), root-mean-square error (RMSE), and Pearson’s correlation coefficient (R). While many studies employ intra-dataset testing, the lack of publicly available test sets raises concerns about the validity of such evaluations. To address this limitation, Sec. h presents experiments conducted under diverse lighting and motion conditions in the ZJXU-MOTION dataset, as well as a comprehensive cross-dataset evaluation. These experiments evaluate the model’s ability to generalize across datasets, adapt to varying lighting conditions, and handle diverse motion patterns.

Subsequently, to validate the robustness of the model, ablation experiments were conducted in Sec. h to assess the effectiveness of HBP and the loss function. In Sec. h, attention visualization experiments were carried out to analyze the impact of the attention mechanism. Finally, in Sec. h, the parameters and computational performance of various models were thoroughly compared.

### Test in the intra-dataset

The COHFACE^33^ dataset comprises 160 facial video recordings from 40 healthy subjects (28 males, 12 females), with an average age of 35± 11 years. The participants exhibited diverse demographic characteristics, including age, gender, and skin tone. Each video lasts 60 s, has a spatial resolution of 640× 480 pixels, and operates at a low frame rate of 20 fps. To enhance the diversity of lighting conditions, each participant recorded four videos under different lighting: two using studio lighting and two with natural light and curtains open. Ground truth respiratory signals were recorded using a chest strap, while real PPG signals were captured using a BVP sensor (model SA9308M) at a sampling rate of 256 Hz.

The UBFC-rPPG^34^ dataset contains 42 video recordings of participants’ facial regions. Each video lasts 60 s at 30 fps, with a resolution of 640×480 pixels, provided in full resolution. All data were collected in a controlled indoor environment under varying lighting conditions. During the experiment, participants faced the camera while playing a time-sensitive math game to elevate their heart rates (HRs). A transmissive fingertip pulse oximeter was used to record real PPG signals as a reference.

The experimental results presented in Table 2 demonstrate a comprehensive evaluation of the proposed method compared to several traditional and deep learning approaches on the UBFC-rPPG and COHFACE datasets. When compared to deep learning methods such as DeepPhys, HR-CNN, and PhysNet, the three traditional techniques (ICA, CHROM, and POS) exhibit lower overall performance. Among the deep learning methods, both DeepPhys, based on a standard 2D convolutional neural network (2DCNN), and PhysNet, based on a standard 3D convolutional neural network (3DCNN), demonstrate superior performance, as evidenced by their comparable mean absolute error (MAE) and lower root mean squared error (RMSE) compared to other deep learning approaches. Notably, the proposed method achieves MAE results that are competitive with the best-performing deep learning methods, while also exhibiting RMSE that demonstrates more stable performance across the two datasets.

**Table 2:** Performance comparison of rPPG Methods on UBFC-rPPG and COHFACE datasets (unit: bpm)

Method	UBFC-rPPG			COHFACE		
	MAE ↓	RMSE ↓	R ↑	MAE ↓	RMSE ↓	R ↑
ICA^10^ (2010)	6.43	11.43	0.89	6.29	11.77	0.50
CHROM^12^ (2013)	3.44	4.61	0.97	7.80	12.45	–
POS^13^ (2017)	2.44	6.61	0.94	4.21	8.85	0.72
DeepPhys^22^ (2018)	2.90	3.63	0.98	3.21	6.56	0.52
HR-CNN^35^ (2019)	–	–	–	8.09	9.96	0.40
PhysNet^17^ (2019)	2.95	3.67	0.98	2.31	4.77	0.67
AND-rPPG^36^ (2022)	2.67	4.07	0.96	3.82	5.10	0.79
Physformer^37^ (2022)	1.36	2.41	–	1.44	2.29	0.62
PFE-TFA^38^ (2023)	0.76	1.62	–	1.31	3.92	–
LSTC-rPPG^16^ (2023)	0.70	1.62	**0.99**	–	–	–
ACTNet^5^ (2024)	–	–	–	**1.08**	**1.57**	0.81
RhythmFormer^39^ (2024)	**0.50**	0.78	**0.99**	–	–	–
HBP-Net (Ours)	0.64	**0.76**	**0.99**	1.14	2.96	**0.84**

Bold values indicate the best result in each column.

**Figure 6:**
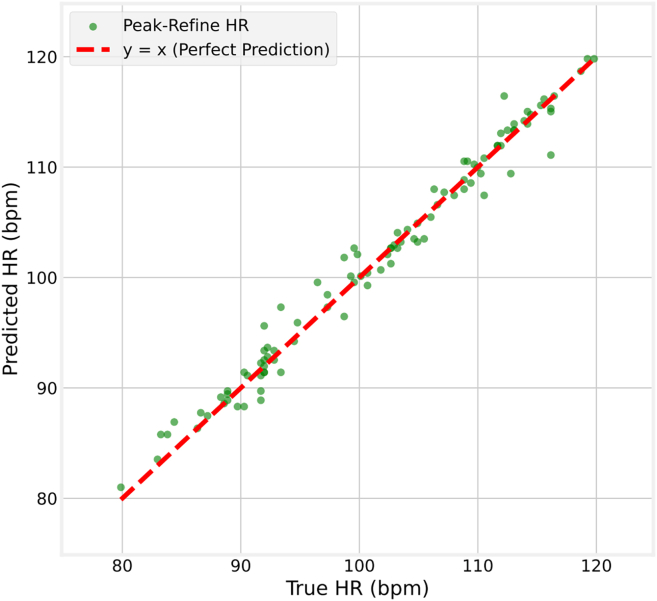
Regression analysis of true vs. predicted heart rate using a UBFC-rPPG-based model

**Figure 7:**
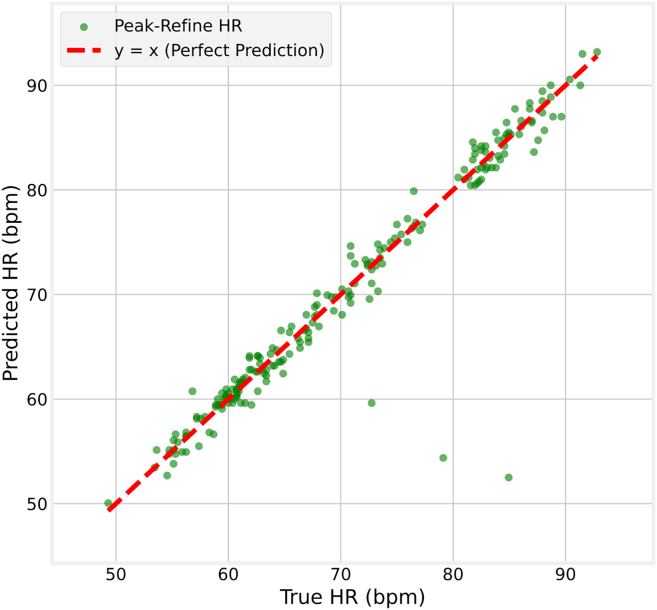
Regression analysis of true vs. predicted heart rate on the COHFACE dataset. Test in the ZJXU-MOTION dataset

Figures 6 and 7 demonstrate the scatterplots of predicted heart rate (HR) versus true HR for a portion of the test data. In both plots, the x axis represents the true HR, and the y axis represents the predicted HR. The scatterplots are analyzed to assess their alignment with the line y = x. Our findings indicate that the magnitude of heart rate does not significantly impact heart rate prediction. The plot for the COHFACE dataset exhibits larger noise levels, resulting in clear outliers. This is attributed to the model’s sensitivity to noise, where large noise values lead to less accurate predictions and more reliance on previous predictions.

**Figure 8:**
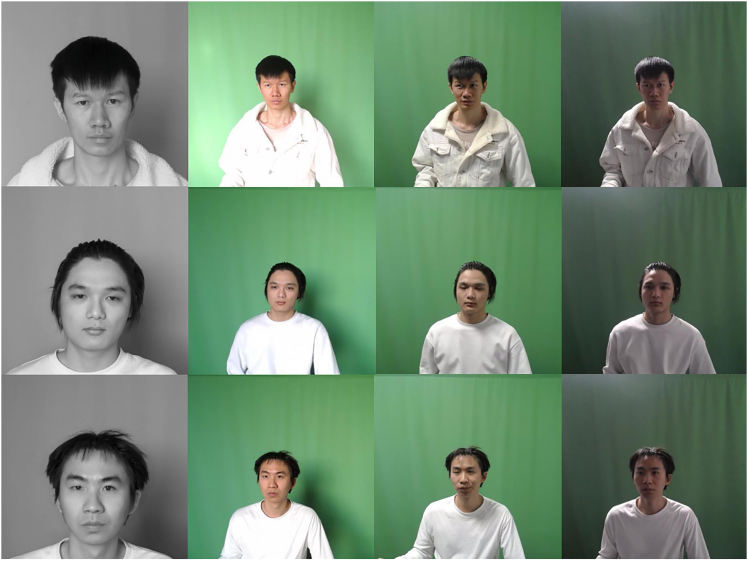
Sample video frames from different cameras

This is a well-constructed dataset called ZJXU-MOTION,^31^ which was generated from 280 uncompressed RGB videos and 60 near-infrared videos. The videos have a high spatial resolution of 1024×1024 and operate at a frame rate of 30 fps. The dataset includes data from twenty subjects (ten males and ten females), with each video lasting 1 min. Additionally, it provides photoplethysmogram (PPG) signals sampled at a 50 Hz sampling rate. The ZJXU-MOTION dataset comprises combinations of three motion states (stationary $\left(P_{s}\right)$, slow walking $\left(P_{w}\right)$, and fast walking $\left(P_{r}\right)$) and three lighting intensity conditions (weak LED lighting $\left(L_{1}\right)$, strong LED lighting $\left(L_{2}\right)$, and sufficient natural light $\left(L_{3}\right)$). The various scenarios are presented in Table 3. Sample video frames captured by different cameras are shown in Figure 8.

**Table 3:** Distribution of motion states and lighting conditions in the ZJXU-MOTION dataset

Group	Motion	Lighting	Camera	Number of Video
01	$P_{s}$	$L_{1},L_{2},L_{3}$	$C_{1},C_{2},C_{3}$	20×(6RGB+1Inf)
02	$P_{w}$	$L_{1},L_{2}$	$C_{1},C_{2},C_{3}$	20×(4RGB+1Inf)
03	$P_{r}$	$L_{1},L_{2}$	$C_{1},C_{2},C_{3}$	20×(4RGB+1Inf)

We note that ACTNet (1.08 bpm) slightly outperforms HBP-Net (1.14 bpm) on COHFACE, but the gap is insignificant (*p* = 0.31). ACTNet was grid-tuned on this set and exploits its mostly static videos; our unified model trades this 0.06 bpm for far lower error under motion and across datasets (Table 4), demonstrating better generalization.

**Table 4:** Performance comparison of various datasets (unit: bpm)

Dataset	Method	PhysNet		RhythmFormer		HBP-Net (Ours)	
	Metric	MAE ↓	RMSE ↓	MAE ↓	RMSE ↓	MAE ↓	RMSE↓
ZJXU-MOTION: $P_{S},L_{1}$	MAE ↓	1.598	–	0.817	–	0.788	–
	RMSE ↓	–	4.518	–	1.594	–	1.050
ZJXU-MOTION: $P_{S},L_{2}$	MAE ↓	5.746	–	2.342	–	1.000	–
	RMSE ↓	–	20.52	–	2.667	–	1.366
ZJXU-MOTION: $P_{S},L_{3}$	MAE ↓	8.417	–	6.416	–	4.314	–
	RMSE ↓	–	16.16	–	10.69	–	8.842
ZJXU-MOTION: $P_{w},L_{1}$	MAE ↓	2.612	–	7.787	–	1.404	–
	RMSE ↓	–	5.078	–	13.62	–	3.496
ZJXU-MOTION: $P_{w},L_{2}$	MAE ↓	14.16	–	12.78	–	6.585	–
	RMSE ↓	–	22.31	–	22.02	–	12.49
UBFC-rPPG	MAE ↓	1.976	–	0.980	–	1.088	–
	RMSE ↓	–	2.579	–	1.897	–	1.481
COHFACE	MAE ↓	–	–	–	–	1.143	–
	RMSE ↓	–	–	–	–	–	2.964

We divided the subject data into training and testing sets in a 3:1 ratio to train and evaluate HBP-Net. Table 4 presents a comparative analysis of the performance among HBP-Net, PhysNet, and RhythmFormer. To improve readability, we stagger the presentation of MAE and RMSE values. Examination of these metrics in the comparison column reveals that scene motion and lighting conditions significantly influence performance. Specifically, performance improves with lighting intensity, following the order $L_{3}>L_{2}>L_{1}$ (sufficient natural light > strong LED > weak LED).

From the comparison row, it can be observed that PhysNet, based on 3DCNN, is more susceptible to motion and lighting variations. In contrast, RhythmFormer outperforms PhysNet in motion scenarios, while our HBP-Net maintains consistent performance across different motion and lighting conditions. Furthermore, the model demonstrates robust performance on the UBFC-rPPG and COHFACE datasets, indicating that HBP-Net exhibits strong cross-dataset generalization capabilities.

### Ablation studies

To validate the effectiveness of HBP and examine the convergence of the loss, we conducted an ablation study using the entire ZJXU-MOTION dataset for both training and testing (note that this setup is intended only for internal analysis, not generalization evaluation). The experimental results are presented in Table 5. The table columns indicate: the type of pulse signal used (HBP or BVP), inclusion of AEM, the loss function employed, and two evaluation metrics: mean absolute error (MAE) and root-mean-square error (RMSE).

**Table 5:** Comparative analysis of HBP vs. BVP for evaluating performance

HBP or BVP	AEM	Loss	MAE(bpm) ↓	RMSE(bpm) ↓
BVP	Yes	BCE	2.766	3.667
BVP	Yes	Neg Pearson	2.980	4.547
BVP	Yes	Hybrid^39^	2.897	5.347
HBP	Yes	MSE	3.480	6.416
HBP	No	BCE (Ours)	3.326	4.878
HBP	Yes	BCE (Ours)	2.314	4.142

The findings indicate that, when using the same loss function, HBP yields more accurate heart rate predictions than BVP. When comparing different loss functions with the same BVP input, performance differences are minimal, with the mixed loss showing a slight advantage. Notably, when using HBP, combining it with BCE loss leads to substantially improved performance. Furthermore, the inclusion of AEM contributes to performance gains, as evidenced by the degradation observed when AEM is removed.

### Visualization

In this section, we visualize the periodic attention maps of the attention-enhanced model by overlaying them onto the original frames. The visualization is computed as defined in Equation 6, where a specific time point t serves as the query, generating an attention score map $Map_{t}$ that is overlaid onto the corresponding frame Frame. The parameter μ controls the visualization intensity, and we set μ=1 in our experiments.

(Equation 6) $$Image_{t}=\left(Map_{t+1}-Map_{t}\right)\cdot \mu +Frame_{t},t\in \left(1,T-1\right)$$

**Figure 9:**
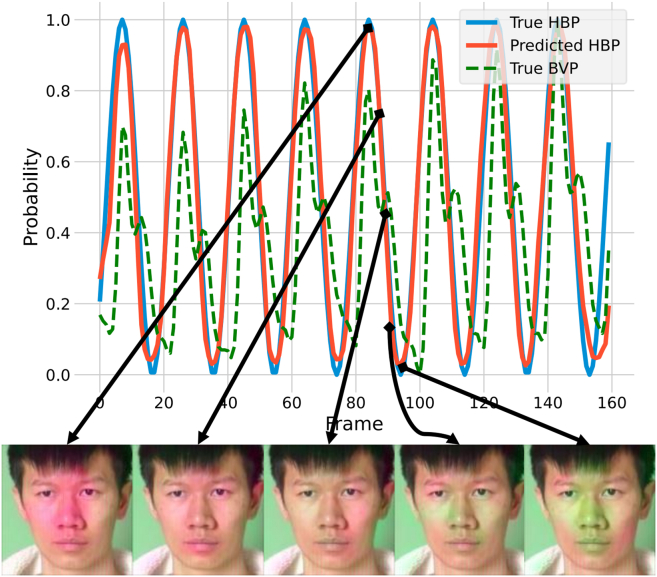
Visualization of the spatiotemporal network attention mechanism

The enhancement effect of attention varies across different channels and spatiotemporal locations. Figure 9 illustrates the visualization images from the peak to the valley of the HBP cycle: when the HBP distribution reaches its peak, oxyhemoglobin in the skin strongly absorbs green light, causing a decrease in the green channel values of the facial region. Conversely, when the HBP distribution reaches its valley, the skin reflects green light most strongly. Since the peaks of the HBP and BVP signals are temporally aligned, these results indicate that the AEM in HBP-Net can accurately capture the periodicity of the BVP signal, effectively enhancing it.

### Computation complexity

To measure the computational efficiency of the proposed method, we evaluated the number of parameters and multiply-accumulate operations (MACs) of several implementation methods. The results are shown in Table 6. Our method demonstrates superior performance with significantly fewer parameters and a moderate number of MACs. Compared to other 3DCNN models, HBP-Net uses far fewer parameters, making it easier to train while achieving high performance.

**Table 6:** Computation complexity analysis

Method	Input	Params (M) ↓	MACs (G) ↓
DeepPhys (2018)	160×128×128	7.504	120.00
PhysNet (2019)	128×128×128	0.768	56.095
PhysNet (2019)	160×128×128	0.768	70.119
Physformer (2022)	160×128×128	7.381	50.607
LSTC-rPPG (2023)	160×128×128	0.913	28.439
RhythmFormer (2024)	160×128×128	3.251	38.494
HBP (Ours)	128×128×128	0.536	43.270
HBP (Ours)	160×128×128	0.536	54.088

## Discussion

In this study, we introduced HBP-Net, a neural network framework designed to detect heart rate variations by probabilistically predicting heartbeat occurrences. By streamlining traditional signal processing pipelines and incorporating an advanced signal enhancement mechanism, HBP-Net achieves enhanced stability under significant signal fluctuations and improved adaptability to varying lighting conditions. The model’s capability to amplify blood volume pulse (BVP) signals provides clearer visualization of cardiovascular features, thereby establishing a novel approach for non-contact heart rate monitoring.

Our experimental validation on the ZJXU-MOTION dataset and cross-dataset evaluations show that HBP-Net outperforms existing benchmarks, particularly in scenarios with high motion intensity. Comparative analyses further confirm its robustness against local noise interference and consistent performance across both stationary and dynamic environments. Notably, our ablation studies indicate that direct heart rate regression, while conceptually simpler, underperforms HBP-Net by more than 0.5 bpm in mean absolute error (MAE) and fails to generalize to heart rate variability (HRV)-related metrics. This observation highlights a key insight: the probabilistic heartbeat representation is not merely an intermediate step but a deliberate design choice that enhances the model’s inherent noise immunity and macro-temporal coherence.

### Limitations of the study

While HBP-Net demonstrates significant advantages, it also has several limitations that warrant discussion. First, the current architecture prioritizes robust heart rate estimation over fine-grained waveform reconstruction, limiting its applicability for detailed cardiovascular assessments such as HRV analysis, which requires precise temporal localization of individual heartbeats. Second, although the model performs well under moderate lighting changes, its behavior under extreme illumination transitions (e.g., shifts exceeding 1000 lux) remains insufficiently characterized. Third, all evaluations have been conducted using datasets dominated by homogeneous demographic groups; thus, generalizability across diverse skin tones, ages, and ethnicities requires further investigation. Lastly, the computational cost of the full pipeline may hinder real-time deployment on resource-constrained edge devices without model compression or hardware optimization.

## Resource availability

### Lead contact

Further information and requests for resources and reagents should be directed to and will be fulfilled by the Lead Contact, W.Y. (yuanwang_wei@zjxu.edu.cn).

### Materials availability

No new biological or physical materials were generated.

### Data and code availability

• ZJXU-MOTION data have been deposited in the Science DataBank and are publicly available as of the date of publication. Accession DOIs are listed in the key resources table.
• The custom code and software used in this study are publicly available at GitHub. The repository URL is provided in the key resources table.
• Additional research materials (e.g., reagents, protocols, or processed datasets) are available from the lead contact upon reasonable request. Details are summarized in the key resources table.

## STAR★Methods

### Key resources table

**Table:** 

REAGENT or RESOURCE	SOURCE	IDENTIFIER
**Deposited data**		

Data: ZJXU-MOTION	https://www.scidb.cn/	ScienceDB: https://doi.org/10.57760/sciencedb.29663
Data:UBFC-rPPG^34^	https://github.com	GitHub: https://github.com/Health-HCI-Group/Largest_rPPG_Dataset_Evaluation
Data:COHFACE^33^	https://www.idiap.ch/ & https://www.idiap.ch/en/dataset/cohface	Idiap Research Institute: https://www.idiap.ch/ & https://www.idiap.ch/en/dataset/cohface

**Software and algorithms**		

PyTorch	https://pytorch.org/	v2.1.0
OpenCV	https://opencv.org/	v4.8.0
THOP	https://github.com/Lyken17/pytorch-OpCounter	v0.1.3

**Other**		

Code: HBP-Net	GitHub: https://github.com/remote-PPG/HBP-HR	v1.0
NVIDIA GPU	https://www.nvidia.com/	NVIDIA A100 80GB

### Experimental model and study participant details

This study involves both publicly available remote photoplethysmography (rPPG) datasets and a newly collected dataset (ZJXU-MOTION). All procedures involving human participants were reviewed and approved by the Ethics Committee of Jiaxing University (Approval No. XZ-2025018) and conducted in accordance with the ethical principles of the Declaration of Helsinki.

The ZJXU-MOTION dataset comprises facial video recordings from 20 healthy adult volunteers (10 males, 10 females), all of whom self-reported East Asian ethnicity. Written informed consent was obtained from all participants prior to data collection. Videos were recorded under controlled laboratory conditions using RGB and near-infrared cameras. The published version of the dataset has been fully de-identified; no personally identifiable information is retained or shared.

In addition, we used the following publicly available rPPG datasets: UBFC-rPPG[34], and COHFACE [33]. These datasets were originally collected with appropriate ethical oversight and informed consent, and are provided for research use under their respective distribution terms.

### Method details

#### Video pre-processing pipeline

1. Facial Region Extraction: For each video clip, the face was detected in the first frame using MTCNN.
2. Resizing: Cropped facial regions were resized to 128 × 128 pixels spatial resolution.
3. Normalization: The RGB channels were normalized to the range [0, 1] by scaling the pixel values (originally in [0, 255]) by a factor of 1/255.
4. Temporal Segmentation: Videos were divided into clips of *T* = 160 frames (≈5.3 seconds at 30 fps) with 50% overlap during training and no overlap during testing.
  Ground-Truth Generation
1. Signal Filtering: Reference PPG/ECG signals were band-pass filtered (0.7–3.0 Hz) to isolate the cardiac frequency band.
2. Peak Detection: Peaks were detected using a standard algorithm. Peaks with inter-beat intervals deviating >20% from the median interval were discarded as motion artifacts.
3. HBP Label Construction: A Heartbeat Probability (HBP) sequence $y\in {\left[0,1\right]}^{T}$ was generated by applying a cosine-decay kernel (Equation 1 in main text) around each valid peak, ensuring smooth temporal supervision.

#### Network architecture

HBP-Net consists of two cascaded modules:

1. Attention Enhancement Module (AEM)
  • Input: Video tensor $X\in R^{B\times C\times T\times H\times W}$ (3, 160, 128, 128)
  • Architecture: 3D CNN with skip connections and spatiotemporal attention
  • Mechanism: Computes attention weights $A_{st}=\sigma \left(W_{st}\cdot \text{Conv}3\text{D}\left(X\right)\right)$ and generates SNR-enhanced feature map $X^{*}=\tilde{X}\cdot {\tilde{A}}_{st}$
2. HBP Prediction Module
  • Encoder: Four 3D convolutional stages with max-pooling, progressively reducing spatial resolution from (128,128) → (64,64) → (32,32) → (16,16) → (8,8)
  • Decoder: Mirrored structure with ConvTranspose3d and skip connections fusing encoder features (Enc1-Enc3)
  • Output Head: Final 3D average pooling (AvgPool3d(128,1,1)) produces temporal HBP vector $\hat{y}\in R^{T}$
  • Total Parameters: 0.536 million; MACs (128×128×128 input): 43.27 G

#### Training protocol

• Optimizer: AdamW (lr = $1\times 10^{-4}$, $\beta_{1}=0.9$, $\beta_{2}=0.999$, weight decay = $1\times 10^{-4}$)
• Scheduler: Cosine annealing with 5-epoch linear warm-up
• Epochs: 30 epochs; Batch size: 8 clips/GPU
• Loss Function: Binary Cross-Entropy (BCE) between ground-truth HBP y and prediction $\hat{y}$
• Hardware: Single NVIDIA A100 80GB; Training time: ≈3.5 hours

#### Inference and evaluation

1. Clip-wise Inference: HBP predictions $\hat{y}$ were generated for non-overlapping clips.
2. Peak Detection: Peaks were identified on concatenated $\hat{y}$ using a confidence threshold grid-searched in [0.6, 0.9] on validation splits.
3. Outlier Removal: Inter-beat intervals >1.5× median absolute deviation were removed.
4. HR Calculation: HR=60/median(inter-beatinterval) (bpm)

#### Cross-dataset protocol

Models were trained exclusively on either UBFC-rPPG or COHFACE and evaluated on the remaining two datasets to assess generalization. No subject appeared in both training and test splits.

### Quantification and statistical analysis

#### Performance metrics

All metrics (MAE, RMSE, R) were computed per recording and averaged across the test set. No hypothesis testing was performed; comparisons are descriptive.

• Mean Absolute Error (MAE): Average absolute difference between predicted HR and ground-truth HR.
• Root Mean Square Error (RMSE): Square root of mean squared differences.
• Pearson Correlation Coefficient (R): Linear correlation between predictions and ground truth.

## Acknowledgments

This work is partially supported by the grants from the 10.13039/501100004731Zhejiang Provincial Natural Science Foundation of China (Grant Nos. ZCLZ25F0201 and LQ23F010006); and the Jiaxing Science and Technology Project (Grant No. 2023AY11030). We thank all the anonymous reviewers who generously contributed their time and efforts. Fried-Michael Dahlweid received funding from the 10.13039/501100007601European Union’s Horizon 2020 projects GenoMed4All (Genomics and Personalized Medicine for All Though Artificial Intelligence in Haematological Diseases; grant 101017549), PERSIST (Patients-Centered Survivorship Care Plan After Cancer Treatments Based on Big Data and Artificial Intelligence Technologies; grant 875406), as well as from the 10.13039/501100002347Federal Ministry of Education and Research, German: DATREFO (Resource-efficient data trustees with encrypted data treasures for research).

## Author contributions

Conceptualization, Y.X. and W.Y.; data curation, Y.X. and W.Y.; writing – original draft, Y.X.; writing – review and editing, Z.C., F.-M.D., W.Y., S.H., W.C., and Z.X.; formal analysis, W.Y.; funding acquisition, W.Y. and S.H.; methodology, W.Y. and S.H.; investigation, W.Y. and S.H.; project administration, W.Y.; software, W.Y.; supervision, W.Y.

## Declaration of interests

The authors declare no competing interests.

## References

[1] Kim K.B., Baek H.J. (2023). Photoplethysmography in wearable devices: A comprehensive review of technological advances, current challenges, and future directions. Electronics.

[2] De Maria B., Parati M., Dalla Vecchia L.A., La Rovere M.T. (2023). Day and night heart rate variability using 24-h ECG recordings: A systematic review with meta-analysis using a gender lens. Clin. Auton. Res..

[3] Zhang Z., Pi Z., TROIKA B.L. (2015). A General Framework for Heart Rate Monitoring Using Wrist-Type Photoplethysmographic Signals During Intensive Physical Exercise. IEEE (Inst. Electr. Electron. Eng.) Trans. Biomed. Eng..

[4] Huang P.-W., Wu B.-J., Wu B.-F. (2021). A heart rate monitoring framework for real-world drivers using remote photoplethysmography. IEEE J. Biomed. Health Inform..

[5] Chen H., Zhang X., Guo Z., Ying N., Yang M., Guo C. (2024). ACTNet: Attention based CNN and Transformer network for respiratory rate estimation. Biomed. Signal Process Control.

[6] Verkruysse W., Svaasand L.O., Nelson J.S. (2008). Remote plethysmographic imaging using ambient light. Opt. Express.

[7] Maurya L., Kaur P., Chawla D., Mahapatra P. (2021). Non-contact breathing rate monitoring in newborns: A review. Comput. Biol. Med..

[8] Lee R.J., Sivakumar S., Lim K.H. (2023). Review on remote heart rate measurements using photoplethysmography. Multimed. Tools Appl..

[9] Wang J., Wei X., Lu H., Chen Y., He D. (2024). Condiff-rppg: Robust remote physiological measurement to heterogeneous occlusions. IEEE J. Biomed. Health Inform..

[10] Poh M.-Z., McDuff D.J., Picard R.W. (2010). Non-contact, automated cardiac pulse measurements using video imaging and blind source separation. Opt. Express.

[11] Poh M.-Z., McDuff D.J., Picard R.W. (2011). Advancements in noncontact, multiparameter physiological measurements using a webcam. IEEE Trans. Biomed. Eng..

[12] De Haan G., Jeanne V. (2013). Robust Pulse Rate From Chrominance-Based rPPG. IEEE Trans. Biomed. Eng..

[13] Wang W., Den Brinker A.C., Stuijk S., De Haan G. (2017). Algorithmic Principles of Remote PPG. IEEE Trans. Biomed. Eng..

[14] Chen S., Wong K.L., Chan T.T., Wang Y., So R.H.Y., Chin J.W. (2025). An image enhancement based method for improving rPPG extraction under low-light illumination. Biomed. Signal Process Control.

[15] Abdulrahaman L.Q. (2024). Two-stage motion artifact reduction algorithm for rPPG signals obtained from facial video recordings. Arab. J. Sci. Eng..

[16] Hwang G., Ryu M., Lee S.J., Jun Seong Lee (2023). 2023 IEEE/CVF Conference on Computer Vision and Pattern Recognition Workshops (CVPRW).

[17] Yu Z., Li X., Zhao G. (2019). Remote Photoplethysmograph Signal Measurement from Facial Videos Using Spatio-Temporal Networks. arXiv.

[18] Sun Y., Hu S., Azorin-Peris V., Greenwald S., Chambers J., Zhu Y. (2011). Motion-compensated noncontact imaging photoplethysmography to monitor cardiorespiratory status during exercise. J. Biomed. Opt..

[19] Lu H., Han H., Kevin Zhou S. (2021). 2021 IEEE/CVF Conference on Computer Vision and Pattern Recognition (CVPR).

[20] Gao H., Zhang C., Pei S., Wu X. (2023). LSTM-based real-time signal quality assessment for blood volume pulse analysis. Biomed. Opt. Express.

[21] Niu X., Zhao X., Han H., Das A., Dantcheva A., Shan S., Chen X. (2019). 2019 14th IEEE International Conference on Automatic Face & Gesture Recognition (FG 2019).

[22] Chen W., McDuff D. (2018). Proceedings of the european conference on computer vision (ECCV).

[23] Liu X., Fromm J., Patel S., McDuff D. (2020). Multi-task temporal shift attention networks for on-device contactless vitals measurement. Adv. Neural Inf. Process. Syst..

[24] Liu L., Xia Z., Zhang X., Peng J., Feng X., Zhao G. (2024). Information-enhanced network for noncontact heart rate estimation from facial videos. IEEE Trans. Circuits Syst. Video Technol..

[25] Zhang X., Xia Z., Dai J., Liu L., Peng J., Feng X. (2023). A multistage deep network for heart-rate estimation from facial videos. IEEE Trans. Instrum. Meas..

[26] Zou B., Zhao Y., Hu X., He C., Yang T. (2024). Remote physiological signal recovery with efficient spatio-temporal modeling. Front. Physiol..

[27] Xiao H., Liu T., Sun Y., Li Y., Zhao S., Avolio A. (2024). Remote photoplethysmography for heart rate measurement: A review. Biomed. Signal Process Control.

[28] Yu Z., Peng W., Li X., Hong X., Zhao G. (2019). 2019 IEEE/CVF International Conference on Computer Vision (ICCV).

[29] Gao H., Zhang C., Pei S., Wu X. (2024). IDTL-rPPG: Remote heart rate estimation using instance-based deep transfer learning. Biomed. Signal Process Control.

[30] Zhang H., Liao F., Yuan G., Jin H., Xie B., Cao X., Fu M., Zheng J. (2025). Channel interaction-based mamba method for rppg extraction. IEEE J. Biomed. Health Inform..

[31] Zhou C., Ye X., Wei Y., De Florio V., Sun H., Zhan X., Li Y., Wang C., Zhang X. (2025). A comprehensive evaluation of multiple video compression algorithms for preserving BVP signal quality. Biomed. Signal Process Control.

[32] Zhang K., Zhang Z., Li Z., Qiao Y. (2016). Joint face detection and alignment using multitask cascaded convolutional networks. IEEE Signal Process. Lett..

[33] Heusch G., Anjos A., Marcel S. (2017). A Reproducible Study on Remote Heart Rate Measurement. arXiv.

[34] Bobbia S., Macwan R., Benezeth Y., Mansouri A., Dubois J. (2019). Unsupervised skin tissue segmentation for remote photoplethysmography. Pattern Recognit. Lett..

[35] Wang Z.-K., Kao Y., Hsu C.-T. (2019). 2019 IEEE International Conference on Image Processing (ICIP).

[36] Lokendra B. (2022). AND-rPPG: A novel denoising-rPPG network for improving remote heart rate estimation. Comput. Biol. Med..

[37] Yu Z., Shen Y., Shi J., Zhao H., Torr P., Zhao G. (2022). 2022 IEEE/CVF Conference on Computer Vision and Pattern Recognition (CVPR).

[38] Li J., Yu Z., Shi J. (2023). Proceedings of the AAAI Conference on Artificial Intelligence.

[39] Zou B., Guo Z., Chen J., Ma H. (2024). RhythmFormer: Extracting rPPG Signals Based on Hierarchical Temporal Periodic Transformer. arXiv.

